# Case Report: Post obstructive pulmonary edema (POPE) Type II following elective adenotonsillectomy requiring novel use of high frequency oscillatory ventilation (HFOV)

**DOI:** 10.12688/f1000research.16044.1

**Published:** 2018-08-31

**Authors:** Joanna J. Moser, Meghan O'Connell, Debbie L. McAllister

**Affiliations:** 1 Department of Anesthesiology, Perioperative and Pain Medicine, University of Calgary, Foothills Medical Centre, Calgary, Alberta, T2N 2T9, Canada; 2 Department of Anesthesiology, Pain, and Perioperative Medicine, Royal Jubilee and Victoria General Hospitals, Victoria, British Columbia, V8R 1J8, Canada; 3 Department of Pediatric Anesthesia, Alberta Children’s Hospital, Calgary, Alberta, T3B 6A8, Canada

**Keywords:** General Anesthesia, High Frequency Oscillation Ventilation, Pulmonary Edema, Sleep Apnea, Obstructive Tonsillitis

## Abstract

This case report describes a previously healthy 14 year-old patient undergoing elective outpatient adenotonsillectomy that was complicated by acute postoperative pulmonary edema requiring 12 hours of high frequency oscillatory ventilation (HFOV) support.  We describe the clinical findings that led us to this rare diagnosis and management of post obstructive pulmonary edema (POPE) Type II, a rare but recognized complication following the surgical relief of an upper airway obstruction. This case is unique in that no previously published case report or review of POPE Type II has described the need for HFOV support.

## Introduction

This case report describes a pediatric patient undergoing elective adenotonsillectomy complicated by acute postoperative pulmonary edema Type II (POPE II) requiring twelve hours of high frequency oscillatory ventilation (HFOV) support. We describe the case in detail including management decisions and the broad differential diagnosis that was considered for acute bilateral pulmonary edema and discuss the rationale for this case being diagnosed as a rare case of POPE Type II. This case is unique in that no previously published case report or review of POPE II has described the need for HFOV support. This case description was written only after informed consent was obtained from the patient’s parent/legal guardian.

## Case description

 A previously healthy 14 year-old Caucasian female patient weighing 44 kg underwent an elective outpatient adenotonsillectomy for an initial diagnosis of recurrent tonsillitis. General anesthesia was accomplished with a sevoflurane inhalational induction supplemented with intravenous propofol (2 mg kg ^-1^), morphine (0.1 mg kg ^-1^), dexamethasone (0.2 mg kg ^-1^) and ondansetron (0.1 mg kg ^-1^) after intravenous access was established. Direct laryngoscopy revealed a grade 1 Cormack Lehane view of her airway with moderate to large tonsils and an appropriately sized cuffed endotracheal tube was placed without difficulty. Anesthesia was maintained with 1.0 minimum alveolar concentration (MAC) of sevoflurane and she was ventilated with a tidal volume of 6 mL kg ^-1^, positive end-expiratory pressure (PEEP) of 4 cmH _2_0 and fraction of inspired oxygen concentration (FiO _2_) of 0.3. Her preoperative hemoglobin was 136 g L ^-1^ with a hematocrit of 0.4 L L ^-1^. The surgery was complicated by a brisk arterial bleed with an estimated intraoperative blood loss of 600 mL. The patient was resuscitated with 500 mL Pentaspan and 1000 mL Lactated Ringers intraoperatively (for a total of 34 mL kg ^-1^) and she remained hemodynamically stable throughout the surgery. At the conclusion of the surgery, the patient was extubated fully awake with an oxygen saturation (SpO2) of 99% and transferred uneventfully fully monitored on 6 L min ^-1^ blow-by oxygen to the post anesthetic care unit (PACU) where there was one-to-one nursing care.

**Figure 1.**
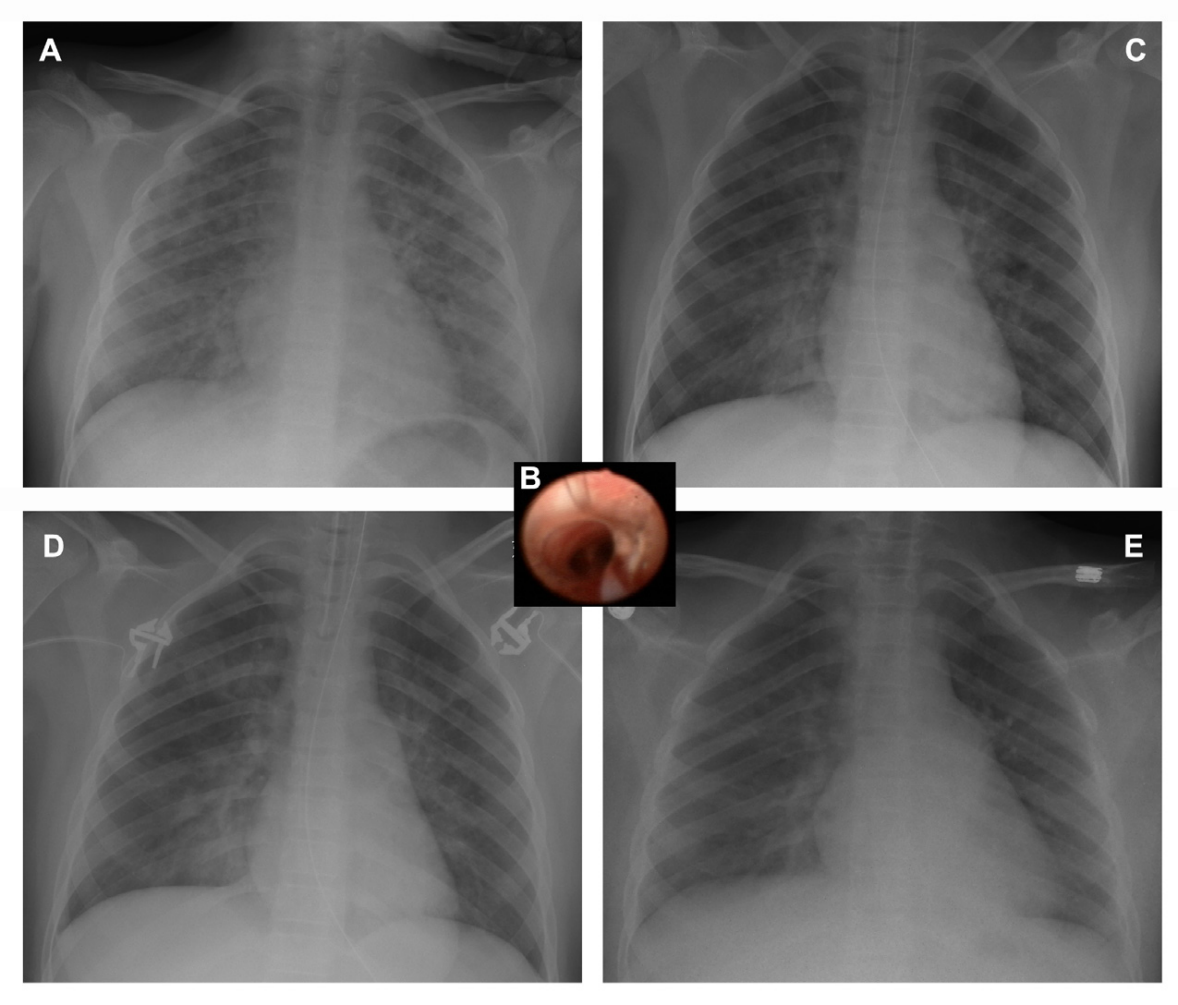
Chest radiographic images of a fourteen year-old patient. Onset of pulmonary edema at hour 0 (panel **a**) and progress at hour 10 (panel **c**), hour 17 (panel **d**) and resolution of post obstructive pulmonary edema at hour 37 (panel **e**). Intraoperative bronchoscopy at hour 1.5 showing pulmonary edema (panel **b**).

On initial assessment in the PACU, she was alert and oriented and talking in full sentences on arrival with an SpO2 of 95% on 10 L min ^-1^ supplemental oxygen by facemask. At this time, bright red blood was suctioned from her oropharynx. Over the course of the next 45 minutes she began spitting up pink frothy sputum and her SpO2 could not be kept above 92% despite supplemental oxygen at 15 L min ^-1^. Throughout her PACU stay, a further 250 mL of blood loss was recorded by nursing. The patient was treated with a total of 4 mg (0.09 mg kg ^-1^) of intravenous morphine for throat pain. During this 45 minute period, the patient was talking to the PACU staff, and there was no documented observation of the patient obstructing their airway. Fifty minutes after arrival in the PACU, the Staff Anaesthesiologist of record was notified of the above course of events in the PACU. A clinical examination revealed bilateral crackles and a lethargic patient requiring continuous positive airway pressure (CPAP) to maintain an SpO2 of 92%. A prompt arterial blood gas (ABG) was obtained, demonstrating a respiratory acidosis (pH 7.24/pCO _2_ 55 mmHg/pO _2_ 101 mmHg, calculated bicarbonate of 24 mmol L ^-1^ and base excess of -4mmol L ^-1^) with a hemoglobin of 79 g L ^-1^. Chest x-ray (CXR) revealed bilateral pulmonary edema ( Figure 1a). 

The patient was taken emergently to the operating room where she had an uneventful rapid sequence re-intubation and surgical hemostasis was confirmed. An intraoperative bronchoscopy demonstrated pink frothy secretions consistent with pulmonary edema distal to the endotracheal tube, with no evidence of aspiration or frank blood in the airways ( Figure 1b). The distal airways were patent and otherwise normal. At this time she had persistent tachycardia up to 130 beats per minute and had intermittent hypotension with systolic blood pressure less than 100 mmHg. Hemodynamics stabilized with one unit of packed red blood cells (273 mL). Post transfusion hemoglobin was 101 g L ^-1^ and furosemide (10 mg; 0.23 mg kg ^-1^) was given to compensate in the event of volume overload or heart failure. The ABG continued to show a respiratory acidosis (pH 7.20/pCO _2_ 65 mmHg/pO _2_ 75 mmHg, calculated bicarbonate of 25 mmol L ^-1^ and base excess of -3 mmol L ^-1^) with an SpO _2_ of 95%, positive end-expiratory pressure (PEEP) 9 cmH _2_0, FiO _2_ of 1.0 and end tidal CO _2_ (ETCO2) of 42 mmHg.

The patient was transferred from the operating room directly to the pediatric intensive care unit (PICU). A bedside transthoracic echocardiogram demonstrated normal left ventricle and right ventricle function, with an estimated left ventricular ejection fraction (LVEF) of 56% and an estimated right ventricular systolic pressure (RVSP) of 30–40 mmHg. At that point, she required manual ventilation to enable adequate oxygenation of non-compliant lungs. Her PaO _2_/FiO _2_ (P/F) ratio ranged from 87 to 156 during the first two hours in the PICU on a conventional ventilator (Maquet Critical Care SERVO-I ventilator system) however, the amount of PEEP required to provide adequate oxygenation/ventilation exceeded 12 mmHg resulting in a peak inspiratory pressure (PIP) greater than 40 cmH _2_0. Forty minutes after arrival in the PICU the patient was placed on a high frequency oscillatory ventilator (HFOV) with a mean airway pressure of 28 cmH _2_O, amplitude of 70 cmH _2_O, power of 5, and FiO _2_ of 1.0, SpO _2_ 100%. She was administered a prophylactic dose of broad-spectrum antibiotics to treat any potential infectious cause to her presentation. Over the next 12 hours the HFOV ventilator settings were titrated down as serial CXRs showed improvement ( Figure 1c and 1d). She was transitioned to a conventional ventilator 13 hours after her arrival in the PICU on postoperative day one. She was extubated to CPAP (8 cmH _2_O) 12 hours later and her CXR showed improvement of lung injury ( Figure 1e). After a further 12 hours of gradual tapering of CPAP therapy, the patient was transferred to the ward with humidified 15 L min ^-1^ blow-by oxygen on postoperative day two. Her ABG on 15 L min ^-1^ blow-by oxygen was pH 7.34/pCO _2_ 45 mmHg/pO _2_ 91 mmHg, calculated bicarbonate of 24 mmol L ^-1^ and base excess of -3 mmol L ^-1^). On postoperative day three, she was discharged home without any respiratory issues and not requiring the use of oxygen therapy. Antibiotic therapy was not continued as her infectious work up was negative. While in the PICU, her parents were questioned in further detail about the patient’s medical history including a comprehensive review of systems given the postoperative course of events. It was at this time the parents revealed for the first time that the patient had an ongoing history of night-time snoring, features consistent with obstructive sleep apnea from enlarged tonsils and adenoids. 

## Discussion

This case report describes a patient who went into acute bilateral pulmonary edema. The differential diagnosis for this presentation is broad, and could include volume overload, acute myocardial dysfunction, acute respiratory distress syndrome (ARDS) secondary to aspiration or infection, transfusion related lung injury (TRALI), or post obstructive pulmonary edema (POPE) Type I or II. This discussion will outline the rationale for this case being diagnosed as a rare case of POPE Type II. 

Volume overload in this patient is unlikely, as she was otherwise healthy and intraoperative fluid resuscitation totalled 34 mg kg ^-1^ (23 mL kg ^-1 ^crystalloid and 11 mL kg ^-1 ^colloid) for resuscitation of an estimated 13.6 mL kg ^-1 ^acute blood loss, with ongoing losses postoperatively (5.7 mL kg ^-1^) for an estimated 28% total blood volume loss. Overall there was a negative fluid balance based on preoperative fasting status and calculated fluid deficit at the end of the first operation. The intraoperative bronchoscopy did not demonstrate any evidence of foreign material (blood or gastric contents) in the airways, lowering the likelihood of an aspiration event as a potential cause for her symptoms. Cardiac dysfunction was excluded as a cause for her respiratory failure with a normal transthoracic echocardiogram study that showed no regional wall motion abnormalities, normal heart valve function, and a normal LVEF. TRALI was excluded as a potential diagnosis given that the transfusion of packed red blood cells was initiated after she was recognized to be in pulmonary edema. Further, the diagnostic criteria of TRALI include new onset of pulmonary infiltrates within six hours after exposure to blood products. In addition, TRALI typically takes multiple days to resolve before clinical improvement ^1^. Finally, the patient did not obstruct at any time during the induction of or emergence from anesthesia, nor did she obstruct her airway in the PACU. Therefore the likelihood of post obstructive pulmonary edema associated with an acute upper airway obstruction causing negative pressure pulmonary edema (POPE type I) was an unlikely diagnosis in this patient given that an obstructive event was not witnessed in a fully observed and monitored patient environment.

Pulmonary edema is a potentially life threatening complication of acute airway obstruction which develops rapidly and often without warning ^2^. POPE Type II is a rare complication that follows surgical relief of a chronic upper airway obstruction, which can occur with hypertrophied adenoids and tonsils ^3^. It has been recognized for decades that upper airway obstruction events can lead to pulmonary edema and right heart failure ^4– 6^. Fluid balance in the lungs is determined by pleural pressures, cardiorespiratory interactions, hydrostatic and oncotic pressure and pulmonary capillary permeability. Over time, breathing against resistance (the Muller manoeuvre) causes wide swings in intrathoracic pressure, which combined with neurohumoral effects of hypoventilation and hypercarbia can predispose the alveoli to edema. The effects of alveolar hypoventilation can lead to increases in pulmonary artery pressure, which in turn can lead to right heart dysfunction and failure. These effects can be reversible with the removal of the airway obstruction ^4^.

With the sudden relief of a chronic upper airway obstruction, such as through surgical removal of hypertrophied tonsils and adenoids or other lesions, the intrinsic PEEP generated by these lesions is lost and the balance between these factors is upset causing the rare presentation of POPE Type II ^7, 8^. The alveoli can flood with interstitial fluid causing acute pulmonary edema. What makes our case unique is that there have been no other reports to our knowledge in the pediatric or adult literature that describe the need for HFOV in order to ventilate patients and allow for the resolution of pulmonary edema associated with POPE Type II. Certainly, in a systematic review and meta-analysis of adult ARDS patients, pooled results suggest that HFOV improves oxygenation, reduces the risk of treatment failure (such as refractory hypoxemia, hypercapnea, hypotension, or barotrauma) and reduces 30-day mortality compared with conventional mechanical ventilation ^9^. Documented cases of POPE Type II have typically resolved after brief supportive care with supplemental oxygen, CPAP, or brief periods of conventional ventilation with PEEP 4 – 8 cmH _2_0 ^2– 5, 10^. In this patient, the level of PEEP and overall airway pressure required to maintain oxygenation and treat the respiratory acidosis was higher than the conventional ventilators available at our institution (Maquet Critical Care SERVO-I ventilator system) could provide. This case highlights that prompt, supportive management, including HFOV, needs to be initiated immediately and that POPE Type II should be considered in the management of acute pulmonary edema post adenotonsillectomy.

## Data availability

All data underlying the results are available as part of the article and no additional source data are required.

## Ethics

Written informed consent for publication of their clinical details and/or clinical images was obtained from the parent/guardian of the patient.
