# Supplementary material for: Case Report: Post obstructive pulmonary edema (POPE) Type II following elective adenotonsillectomy requiring novel use of high frequency oscillatory ventilation (HFOV)
Source: F1000Res. 2018 Aug 31;7:1373. [Version 1] doi: 10.12688/f1000research.16044.1 (PMC6325615; doi:10.12688/f1000research.16044.1)
Supplement: Peer review history

# Peer review: Referee response for version 1

**DOI:** 10.5256/f1000research.17522.r41573

**Published:** 2019-01-07

**Authors:** Andrea Gentili

**Affiliations:** 1 Department of Pediatric Anesthesia and Intensive Care, Sant'Orsola Malpighi Hospital, University of Bologna, Bologna, Italy

The authors describe a case of an acute postoperative pulmonary edema Type II (POPE II) that required high frequency oscillatory ventilation (HFOV) support in a 14-year-old girl undergoing elective adenotonsillectomy.

 The manuscript is interesting, well presented and documented. The discussion is well articulated and appropriate.

 The clinical case demonstrates how even an adenotonsillectomy surgery can lead to major complications such as intraoperative hemorrhage and postoperative pulmonary edema.

 My considerations and suggestions for the authors are:

- The authors could specify which anamnestic elements they used in the preoperative evaluation of the girl's OSA condition.
- Why was the general anesthesia accomplished with a sevoflurane inhalational induction supplemented with intravenous propofol and morphine, when at the patient's age an exclusively intravenous induction could be performed? Was this anesthetic choice made on suspicion of a high airway obstruction from very enlarged tonsils?
- Did the transition from conventional ventilation with a PIP greater than 40 cmH _2_0 to HFOV with a MAP of 28 cmH _2_O involve any cardiovascular changes?
- During HFOV ventilation, for how many hours was the MAP set at 28 cmH _2_O?

 Based on my level of expertise in the subject matter, I believe the manuscript can be indexed.

I have read this submission. I believe that I have an appropriate level of expertise to confirm that it is of an acceptable scientific standard.

# Peer review: Referee response for version 1

**DOI:** 10.5256/f1000research.17522.r37820

**Published:** 2018-09-03

**Authors:** Lynn Martin

**Affiliations:** 1 Department of Anesthesiology and Pain Medicine, Seattle Children's Hospital, University of Washington School of Medicine, Seattle, WA, USA

This is a case report of a 14 yo female developing severe POPE type II following elective tonsillectomy.  The severity of the edema required treatment with HFOV. The case report is well written and referenced. I have few questions or suggestions for the authors:

- I believe the manuscript would be enhanced if more details regarding what screening questions for OSA were or were not asked in the pre-operative period.
- Why are pre-operative hemoglobin and hematocrit obtained?
- Why did it take 45 minutes to notify the staff anesthesiologist of the ongoing blood loss of 250 mL?
- Why was morphine given to a patient in respiratory distress?
- The discussion is excellent and complete. My only suggestion would be to move the second paragraph to the end of the discussion. Thus you are highlighting the unique features of this case report first and finishing be ruling out other potential causes.

I have read this submission. I believe that I have an appropriate level of expertise to confirm that it is of an acceptable scientific standard.
